# Surface Treatment of Polymeric Materials Controlling the Adhesion of Biomolecules

**DOI:** 10.3390/jfb3030528

**Published:** 2012-08-07

**Authors:** Fabienne Poncin-Epaillard, Tjasa Vrlinic, Dominique Debarnot, Miran Mozetic, Arnaud Coudreuse, Gilbert Legeay, Benaïssa El Moualij, Willy Zorzi

**Affiliations:** 1 Département Polymères, Institut des Molécules et Matériaux du Mans, LUNAM Université, UMR Université du Maine—CNRS No. 6283, Colloïdes et Interfaces, Avenue Olivier Messiaen Le Mans Cedex 72085, France; Email: tjasa.vrlinic.etu@univ-lemans.fr (T.V.); Dominique.Debarnot@univ-lemans.fr (D.D.); 2 Jozef Stefan Institute, Jamova cesta 39, Ljubljana 1000, Slovenia; Email: miran.mozetic@ijs.si; 3 CTTM, 20 rue Thalès de Milet, Le Mans 72000, France; Email: acoudreuse@cttm-lemans.com (A.C.); gilbert.legeay@neuf.fr (G.L.); 4 Centre de Recherche sur les Protéines Prion, Institut de Pharmacie, B36, No. 1 avenue de l’Hôpital, Liège 4000, Belgium; Email: willy.zorzi@ulg.ac.be (W.Z.); b.elmoualij@ulg.ac.be (B.E.M.)

**Keywords:** surface, polymer, modification, bioadhesion, biomolecules

## Abstract

This review describes different strategies of surface elaboration for a better control of biomolecule adsorption. After a brief description of the fundamental interactions between surfaces and biomolecules, various routes of surface elaboration are presented dealing with the attachment of functional groups mostly thanks to plasma techniques, with the grafting to and from methods, and with the adsorption of surfactants. The grafting of stimuli-responsive polymers is also pointed out. Then, the discussion is focused on the protein adsorption phenomena showing how their interactions with solid surfaces are complex. The adsorption mechanism is proved to be dependent on the solid surface physicochemical properties as well as on the surface and conformation properties of the proteins. Different behaviors are also reported for complex multiple protein solutions.

## 1. Introduction

Biomaterials are defined as materials that constitute parts of medical implants, devices and disposables are used in medicine, surgery, dentistry and veterinary medicine, as well as in every aspect of patient health care. A material is defined as a biomaterial in respect of some technical and economic rules described in the European Council Resolution of May 1985. The core legal framework of the European law is divided into three directives: No. 90/385/EEC regarding the active implantable medical devices No. 93/42/EEC dealing with medical devices and No. 98/79/EC focused on *in vitro* diagnostic medical devices. A more academic definition of biomaterials is proposed by Williams in 1987, modified in 2003 [1,2] and also by Dee *et al*. [3]. Most of these materials are chosen depending on the final characteristics, however their surfaces are often not compatible with the biological tissues and organisms to which they are exposed [4,5]. Major risks are immunological or inflammatory reactions; there should be no change of plasma enzymes and proteins when they are in contact with living material inside or outside the human body, there should be an absence of toxic and carcinogenic products, no deterioration of tissue and materials and an absence of immune factors responsible for thrombosis and obstructing the flow of blood through the circulatory system [6]. Materials taking into consideration the above mentioned statements can be referred to as “biocompatible” materials and many studies are dedicated to the research of new biocompatible materials due to the increasing number of artificial substitutes every year. Nevertheless, the biocompatibility stays a complex phenomenon, which is still not completely understood due to the lack of knowledge of *in vivo* conditions; and caution should be taken in defining a biomaterial as biocompatible, since the applications of these materials are specific. A biomaterial that is biocompatible or suitable for one application may not be biocompatible in another one [7]. Therefore, the composition and configuration of surfaces and biomolecules should be taken into account.

## 2. Fundamental Interactions between Surfaces and Protein

### 2.1. Protein Structure and Properties

**Figure 1:**
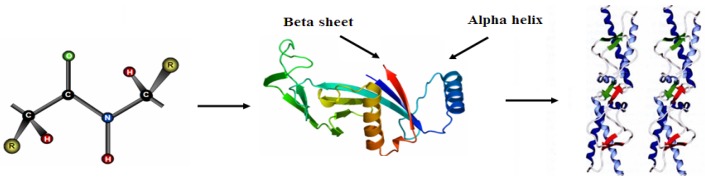
Structure of protein: (**a**) primary structure (peptide bond); (**b**) tertiary structure (composed of secondary structure presenting α-helixes and β-sheets); (**c**) quaternary structure (aggregation of subunits formed in tertiary structure).

Proteins are linear polymers formed by linking the carboxyl group of one amino acid to the α-amino group of another acid with a peptide bond. This primary structure spontaneously folds to regular secondary structures, α helixes or β sheets. The coiled structure is stabilized by intra-chain hydrogen bonds between NH and CO groups, while sheets are stabilized by hydrogen bonding between polypeptide strands (Figure 1). 

The folding of secondary structures into tertiary structures corresponding to spatial arrangement of amino acids [8] enables the formation of active sites able to perform biological functions. In general, proteins fold themselves into globular or fibrous forms. Globular proteins are compactly folded, while the filamentous proteins are elongated. This property will influence their stability and, consequently, the degree of conformational changes upon their adsorption onto the solid surfaces [9]. The folding into tertiary structures is governed by hydrophobic effects, where the interfacial free energy in aqueous solution is decreased by the turnover of non-polar groups inside the aggregate. For this reason, the hydrophobic part is representative of the core of the protein, while polar residues stay on the surface in contact with the solvent. However, in most cases the protein surface is amphiphilic where hydrophobic patches represent one third of the surface coverage. Furthermore, within the same protein, the side chains can be acidic and/or basic, making the polypeptide amphoteric. The overall surface charge of the protein will depend on the pH of the solution [10]. The favored bonding of certain types of residues will also influence the orientation of the biomolecules, which is important for immunological reactions [11,12]. Protein aggregation can occur at all steps in the manufacturing process (cell culture, purification and formulation), storage, distribution and handling of products and results from various factors such as stirring, pH, temperature, ionic strength variations or various interfaces (e.g., air-liquid interface). High protein concentrations can further increase the aggregation. The protein folding and unfolding is an overall process resulting from a cooperative transition. If one part of the protein becomes unstable under exposed conditions, the interaction between this disrupted part and the rest of the protein will be destabilized. Inappropriate protein conformation can result in pathology, as various proteins are related neurodegenerative diseases such as Alzheimer, Huntington and Parkinson disease. The appearance of these diseases is assigned to multimerization of misfolded proteins into insoluble, extra-cellular aggregates and/or intra-cellular inclusions [13].

### 2.2. Proteins and Surfaces

The study of protein-surface interactions gained a lot of interest in the past few decades because these interactions are fundamentally responsible for biocompatibility of the materials. For example, the biological cascade of undesirable reactions like bacterial adhesion or thrombosis can be triggered by protein deposition on the material surface and spontaneous adsorption occurring in order of seconds or minutes after contact. Hence, it is rarely a problem of how to achieve the adsorption of proteins to a surface, but rather how to prevent it. Although the mechanisms of adsorption are not fully understood, there are a few physicochemical characteristics known that influence these phenomena [14,15]: characteristics of the protein (size, stability, concentration, functionalities and protein-protein interactions); the support’s surface free energy (hydrophilic/hydrophobic balance and polarity); the surface charge and related electrostatic interactions; chemical nature, thickness, density and mobility of surface functional groups; micro and nano topography features and roughness; time dependant unfolding; Vroman’s effect; biological surrounding (pH, salts, temperature, *etc*.).

The thermodynamic principles governing the adsorption involve enthalpy and entropic terms that either favor or resist adsorption:

Δ_ads_G = Δ _ads_ H − TΔ_ads_S < 0     (1)

G, H, S and T stand for the Gibbs energy, enthalpy, entropy and temperature of the system, while Δ_ads_ indicates the change in thermodynamic functions of state resulting from the adsorption process. Enthalpy involves intermolecular forces such as van der Waals forces, Coulomb forces, Lewis acid-base forces whereas entropy is based on hydrophobic interactions, conformational entropy and restricted mobility. Adsorption will occur when the change in Gibbs (G) energy becomes negative and there is a net result between attracting and repulsive interactions between material surface, protein molecules and solvent. In general, protein adsorption is believed to occur through several equilibrium states.

First, the proteins are transported to the surface, then they form an energetic boundary layer with attachment and some structural rearrangements. Furthermore, they can be detached and transported away from the surface. During the adsorption step, a protein may rapidly unfold on the surface in order to adapt its energy to the new environment. The adsorption itself happens in microseconds to milliseconds, while relaxation demands much longer periods, ranging from hours to days. In the case of very strong surface-protein affinity, non-equilibrium states can be retained or in other words they irreversibly adsorb to the surface [16]. The first layer of proteins can be adsorbed reversibly or irreversibly, while the second layer tends to adsorb reversibly due to little or no affinity among the molecules themselves caused by water retention [17] or due to their highly regular compact state and uncompleted unfolding caused by their weak flexibility [16]. The adsorption of proteins will also be influenced by surface heterogeneities and the presence of pre-adsorbed molecules [18]. Other important factors that influence bioadhesion are the protein conformational stability in native state, the relative amounts of α helixes and β sheets, and the overall hydrophobicity and electrical charge under the conditions. 

### 2.3. Relationship between Surface Hydrophobicity/Hydrophilicity and Protein Charge

**Figure 2:**
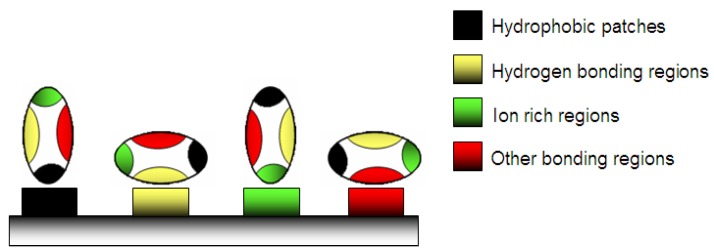
Adsorption of protein on a material surface: various possibilities of physico-chemical adsorption.

**Figure 3:**
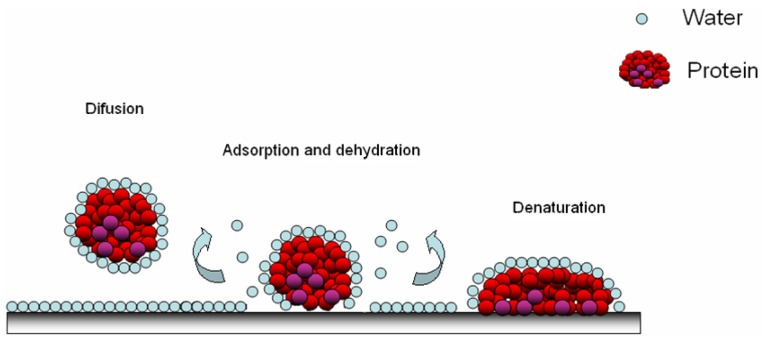
Adsorption of protein on a polymer surface; the diffusion of hydrated protein, adsorption and dehydration possibilities [20].

A weak stable conformation enhances the adsorption on a support by increasing the entropy (Figure 2). Several routes are possible. In contact with hydrophobic surface, the protein adsorbs through its different surface hydrophobic patches. After this, the protein will unfold onto this surface by spreading its hydrophobic core in order to reduce the net hydrophobic surface area of the system in the solvent [19]. The unfolding of the protein is accompanied by the release of water molecules from the interface leading to an entropy gain of the system (Figure 3) [20]. 

**Figure 4:**
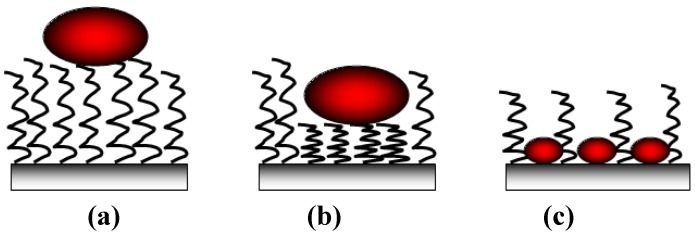
Modes of protein adsorption on polymer brushes on a surface: (**a**) adsorption on the brush; (**b**) attraction and compressive mechanism and (**c**) inclusion into the network brushes.

On the other hand, hydrophilic surfaces interact with the polar and charged functional groups of the protein surface. However in this case, proteins have a much lower tendency to irreversibly unfold on the surface [21]. In both cases, the changes of the hydration state have a large impact. Namely, the water molecules are strongly bonded to proteins and surfaces through electrostatic interactions including hydrogen bonding, rendering the polar groups solvable in water. If the surfaces of the protein and the support are both polar, their hydration is favorable and the retained water molecules at the interface will prevent or diminish adsorption (Figure 3) [22,23]. With polar surfaces, the “hard” proteins will adsorb only if they are electrostatically attracted, while “soft” unstable proteins go through abrupt changes resulting in the increase of conformational entropy large enough to cause their adsorption to otherwise electrostatically repellent surfaces [24]. As the adsorption of proteins on hydrophilic surfaces is mainly due to the Coulomb forces, the design of non-fouling material is often based on polar and uncharged coatings [25] and also on the configuration of the polymeric surface: for example on polyelectrolytes layers [26] or on brushes (Figure 4). In the case of low brush density, proteinic molecules can be intercalated between macromolecular segments of the brushes (Figure 4c). The presence of non-polar patches on the protein surface leads first to the dehydration and consequently to the protein adsorption that is accompanied by the release of bound water molecules from the interface [27]. On hydrophilic surfaces they can adsorb in higher amounts than on hydrophobic surfaces [28].

Generally, both the molecular surface and the protein are electrically charged and surrounded by counter ions that neutralize the surface charge. The formed electrical double layers will enhance the electrostatic attraction if the protein and the surface have opposite charges or the repulsion if the charges are the same. Maximal adsorption occurs when the charge density of the protein matches exactly the one of the surface, which results in a zero net charge at the contact region. The adsorption (to a lower extent) can also be observed on the surfaces that contain the same charge as protein. This indicates that global electrostatic forces affect adsorption but do not necessarily dominate it [29]. The electrostatic interactions are strongly influenced by pH and ionic strength of the solution and, therefore, at low ionic strength, cationic proteins bind to anionic surfaces reversibly. For most proteins, the adsorption on a surface is maximal at their isoelectric points, because the protein-solvent interactions become less favorable and the protein-protein interactions more favorable, due to the zero net charge between them. At the pH far from pI of proteins, the formation of double layers is very likely to be retained due to the electrostatic repulsions, as the charge on protein surfaces becomes more important. On the other hand, proteins are most stable at their pI [30].

### 2.4. Influence of Surface Topography and Roughness on Protein Adsorption

Current trends in research of biomaterials include the study of surfaces with topological features at nano-scale up to 100 nm. The effects of micro scale topography on biomolecule responses are well explored *in vitro* from physicochemical and biological aspects [31,32]. Recently, a few studies revealed the influence of nano-topography on protein adhesion and consequently on cellular responses [33]. The characteristics of topographic features include roughness, curvature and specific geometrical features. Local wettability of the surface is also modulated and induces different degrees of geometrical packaging of the proteins [34]. The overall effect of surface roughness is not clear at this point in time. Some reports suggest that the amount of protein adsorbed is not or very moderately affected by the surface roughness without conformational changes of protein [35]; while other reports reveal high augmentation of the proteins adsorbed and abrupt changes in their conformation upon adsorption [36]. It was stated as well that proteins with dimensions in the same order as the surface roughness are not conformationally altered by the surface, and proteins with dimensions much smaller or much larger change upon adsorption [37]. However, Fournier [38] found no linear relationship between the surface roughness and protein adsorption. Another study [39] also shows dependence of the adsorption not only on substrate topography but also on protein morphology (strand or globules).

### 2.5. Protein Adsorption from Multi-Component Solutions

In complex biological mediums like blood, plasma, cerebrospinal fluid (CSF) or other body liquids, there exist a wide variety of different proteins. As a consequence, these proteins compete among themselves for the adsorption to the exposed surface. An important factor is a mass transfer towards the surface (Vroman effect), which depends on the concentration of individual protein in the solution and is inversely related to its molar molecular weight. Namely, the more concentrated and smaller proteins arrive at the surface first and can be later replaced by larger proteins under the condition that they are able to form stronger interactions with the surfaces [40]. 

## 3. Elaboration of New Biomaterial Surfaces

The adsorption of proteins can be modified by either thermodynamic or kinetic control. Thermodynamic control refers to the alteration of the interaction potential between a protein and the surface by eliminating the attractive interactions between them. Kinetic control can be achieved by slowing down the rate of protein adsorption by high potential barriers for the interaction, for example, by introduction of long range repulsive forces through polymer grafting [40,41,42,43]. Three possible situations of attachment must be avoided: adsorption of protein to the outer edge of the brush due to the protein-brush interaction via van der Waals or electrical double layer attraction; adsorption of proteins upon compression of polymer film for large proteins; adsorption to the surface due to the diffusion of proteins through the brushes to the substrate.

For these reasons, most of the non-fouling surfaces are based on the synthesis of grafted surfaces with highly hydrophilic functional groups. These are in most cases neutral polymers like poly(ethylene glycol) (PEG), polysaccharides, poly(acide methacrylic) (PAAm), poly(2-hydroxyethyl methacrylate) (PHEMA), poly(N-vinyl pyrrolidone) (PVP) and poly(2-methyl-2-oxazoline) (PMOXA) or zwitterionic polymers like phosphoryl chlorine [43,44]. 

Elaboration based on physical sorption of macromolecules can be achieved with the same type of characteristics but presents a deficiency since the added upper layers are solely bonded through relatively weak forces, like electrostatic, hydrogen and van der Walls bonds. For many biomedical applications, materials with a long-term survival and stable coatings with no depletion are necessary. The further discussion of the different elaboration modes, surface functionalization or grafting, will focus on more eco-friendly techniques trying to avoid wet chemistry.

### 3.1. Surface Engineering through One Step Plasma Functionalization

The use of non-equilibrium plasmas for surface modifications is a good alternative to classical organic chemistry reactions [45]. These kinds of modifications are relatively easy and quick, without any toxic solvents, and under soft plasma conditions the ablation of material is negligible. The plasma can be used either to bear various functional groups onto the surface or to grow thin films through plasma polymerization of monomers. However, in the latter case, the coatings are not very strongly attached to the surface. With the former method, non-depositing gases like O_2_, N_2_, CO_2_, CF_4_, He, Ar plasmas induce the attachment of polar or non-polar functional groups like hydroxyl, carbonyl (with O_2_, CO_2_), amine (with N2) and fluorinated groups (with CF4) to the surface. The interactions of such surfaces with a specific surrounding environment are modified. For example, surfaces with grafted non-polar (fluorine) functional groups greatly alter the adhesion of several biomolecules [46]. In order to maintain their optical transmission, PMMA-based contact lenses were plasma-fluorinated and evidence of very low protein adhesion, inflammatory cell growth and cell debris formation were found [47]. On the contrary, polar and hydrophilic surfaces exhibit a higher tendency for the various molecules to adhere and play a significant role in cell adhesion and growth mechanisms [48]. However, this type of surface ages rapidly.

### 3.2. Surface Engineering by a Two-Step Treatment: Surface Activation and Grafting

The introduction of grafted chains can be achieved through various mechanisms: ionic mechanisms, coordination mechanisms and free radical mechanisms, chemical grafting or radiation induced grafting. Firstly, the long-term chemical stability of these layers is much higher due to the covalent bonding to the substrate, secondly there is no or minimal ageing effect of grafted materials, which is especially a problem for hydrophilic modified surfaces in biological environment (water, salts). Finally, the nano (micro)-sized layers have a controlled chemical composition, hydrophilic/hydrophobic balance, roughness and topography. Therefore, the covalent attachment of graft chains to the surface avoids their depletion and by this long term chemical stability is gained [49]. The conformation, thickness and density of grafted polymer chains will depend on the used grafting technique, on the building blocks and the experimental conditions (solvent, pH, T and salt concentrations) [50]. The grafting methods are generally divided into “grafting to” and “grafting from” techniques. 

In the “grafting to” method, the polymer chains are attached by covalent bond to the given surface via chemical reaction between the surface functional groups of the material and the end functionalized group of the polymer backbone [51]. Effective anchoring of polymers can be performed only if appropriate reactive groups are located on the surface of the substrate. First, surfaces are functionalized by different chemical or physical treatments like UV, flame, ion beam treatment, γ irradiation, plasma treatment or by strong oxidizing acids. These kinds of treatment usually lead to formation of oxygen, nitrogen or sulfur containing functional groups on the material surface. In the next step, the functional molecules containing the desired properties are attached to the activated substrate. The advantage of such functionalization is that the molecular weight and chain length of the polymer are well defined. Its negative point is the lower grafting density of the surface due to the steric hindrance and overlapping of the pre-adsorbed polymer molecules on the surface. Additionally, the chain thickness is pre-defined with the length of grafted molecules and cannot be freely adjusted [52]. 

For these reasons, an alternative “grafting from” method is often employed. “grafting from” or so-called surface initiated polymerization (SIP) is based on the formation of thin films through polymerization of monomers from surface bound polymerization initiators [53]. The choice of initiator must be appropriate for the method of polymerization that will be used afterwards. In the case of conventional radical polymerizations (RAFT, ATRP), initiators like peroxide, azo initiators or photo initiators are used. As the chains grow from the surface, the initiators are easily accessible and therefore, high-density polymer brushes can be formed [54]. Many factors can influence the conformation of the anchored chains on the substrate, like the solvent (pH, T °C and salt concentration), type of (co)polymers (monomers), identity and quantity of the functional groups, *etc*. [55,56]. In a good solvent, single reactive side chain groups form brushes, while multiple reactive sites form loops and tails at the surface of a grafted material. In a diluted regime, the polymer chains adopt one of the extreme conformations: Either they appear as a mushroom-like structures in a good solvent or as a pancake-like structure in a bad solvent. The adaptive polymer brushes are able to change their conformation with small changes in the surrounding biological environment, such as light, T (°C), pH and salt concentration [57,58]. The photo responsive-layers are based on the incorporation of photo-active groups like azo-benzene chromophores on the surface that are able to react with UV light. This light source changes the conformation of –N=N– group from *trans* to *cis * isomers which, as a consequence, causes collapse of the chains towards the surface. The response of polymers to temperature is frequently caused by hydrogen bonding groups in the polymer backbone. Below the lower critical solution temperature (LCST), the polymer is usually completely soluble in water; but when the temperature is increased, phase separation occurs and compact globules are formed. One of the most used polymers in thermo-adaptive studies is poly (N-isopropylacrylamide) (PNIPAM), due to the fact that its LCST is close to the human body temperature [56,57,58,59]. At temperatures below the LCST, PNIPAM forms extended structures with a random coil conformation. The hydration of chains is enabled by the hydrogen bond formation between secondary amide hydrogen N–H and carbonyl C=O groups with surrounding water molecules. Nonetheless, when the temperature is increased the hydrophobic interactions predominate and the polymer collapses into globular structures, leading to its precipitation out of water. This transition effect is the result of a temperature induced entropy gain from the dehydration of amide moieties and is completely reversible. This behavior may be associated with the phenomena of undesired protein folding known to cause various fatal disorders, like the accumulation of amyloïd plaques and consequent development of Alzheimer disease [60]. Wu *et al.* [61] observed for the first time a stable single chain to single globule transition in extremely diluted PNIPAM solutions by DLS measurements. The chain density in the globule state was slightly lower than predicted on the basis of a space-filling model, meaning that there was still some water present in the fully collapsed state; the roughness decreased from 127 to 17.9 nm with decreasing temperatures. The measurement with a small increase of temperature displayed two intermediate (crumpled coil and the molten globule) states between the formations of collapsed globules from extended random coils. Upon the adsorption of PNIPAM to the surface, new properties and a new behavior could be induced based on the model of phase behavior of end-grafted polymers exhibiting LCST [62]. The transition was shown to depend on the grafting density and on the molar mass of the chains. In some cases, the transition was negligible or could not even be observed [63]. Introduction of additives to the PNIPAM backbone can also change the polymer LCST. By these modifications, better control of parameters can be achieved that are especially important for *in vivo* applications like temperature responsive membranes, temperature responsive chromatography, immuno-tests, control of drug and growth factor release, tissue engineering, control of the attachment and detachment of the cells, recovery of cultured cells and bio-fouling releasing coating [64]. PNIPAM can also be coupled with various biological molecules (DNA, protein, antibodies) and can be used for the purification of proteins and enzymes. The antibody reacts specifically with the searched antigen and, in the next step the labeled detection antibody is coupled. When the temperature is increased, they precipitate and interact with each other and by this increase the signal. As the signal is multiplied, lower levels of proteins can be detected [65]. The growth of cells on PNIPAM is enhanced above the LCST temperature, where the polymer possesses hydrophobic properties. The cells attach to the surface and proliferate. As the growth support is cooled down, the cells are reversibly released from the surface, without any conformational or functional changes [66]. The same can be applied for reversible attachment and purification of proteins like avidin, BSA, HSA, myoglobin, cyt-C and lysozyme [67,68]. Another class of stimuli-responsive polymers corresponds to the polyelectrolyte. Their structure is almost exclusively dominated by electrostatic reactions. They can change under abrupt alterations of pH, salt concentrations or electric field. Frequently, polyacrylic acid based polymers are selected for this purpose [69]. 

Further development of adaptive surfaces was achieved through grafting of mixed polymer brushes. Besides the importance of functional groups for the specific behavior and the morphology of the surface can be influenced by various ratios of functionalities and solvent properties [70]. The ratio of components is controlled by the time of grafting reaction for each of the components. A similar behavior as for mixed brushes can be observed for block-copolymer brushes grafted on the surface. The great interest in this kind of materials is raised from the fact that the building blocks of di- or tri-copolymers are usually immiscible functional groups, which as a consequence allows them to self-assemble in well organized nanoscale periodic structures. Typically these are spherical, cylindrical or lamellar forms, depending on the composition of the copolymers [71,72].

### 3.3. Surface Engineering through Surfactant Adsorption

**Figure 5:**
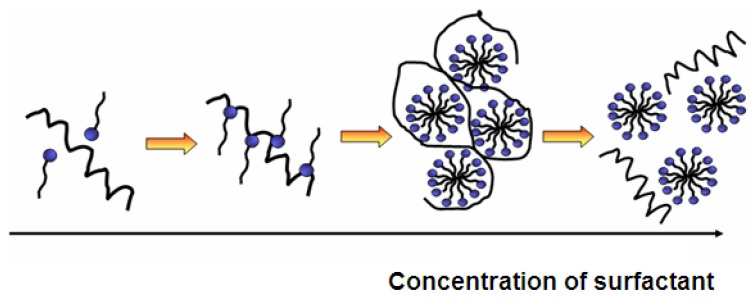
Influence of surfactant concentration on self-assembly of protein and surfactant and formation of mixed micelles.

The adsorption of surfactants on a substrate is mainly determined by two factors: The first one is the interaction of the surfactant with the surface and the second one is the hydrophilicity/hydrophobicity balance. In other words, the hydrophobic chains are not soluble in water and therefore they prefer to adsorb to the surface. The adsorption level is also increased with the augmentation of critical packing parameter (CPP). The CPP demonstrates how tightly surfactants are able to pack at the surface. In aqueous solutions, the high packaging is rather unfavorable and can be artificially increased in several ways. In order to benefit from the fact that hydrophobic interactions lead to higher adsorption, longer and branched or multiple hydrocarbon chains can be used. Additionally, the presence of salts reduces the repulsive electrostatic forces between surface and surfactant, leading to a smaller effective cross-sectional area per surfactant. For this reason, neutral surfactants adsorb much stronger than for example anionic surfactants (Figure 5).

On non-polar surfaces, the hydrophobic chains of the surfactant interact with the surface, while its hydrophilic moieties stay in contact with the solution. The adsorption free energy of surfactants at the hydrophobic surfaces is similar to the micellization free energy of surfactants, and hence the structures on the surface appear like micelles. Contrarily, on polar surfaces surfactants adsorb with their polar component while the non-polar tail stays in the solution. This is only possible with a very low surfactant concentration due to the fact that it is energetically unfavorable to have insoluble chains in the solution. Therefore, with the increase of surfactant concentration, the hydrophobic parts of surfactants in the solution will further adsorb and form bilayers with pre-adsorbed surfactants. The bilayers are formed when there is an extremely strong interaction between surfactant head groups and surface chemical sites. If the interaction strength is moderate, micelles or similar aggregates will be formed. In the case of surfactant adsorption to polar surfaces, micellization occurs considerably below the critical micelle concentration (CMC) of surfactants (ionic and non-ionic) due to surface induced self-assembly [73].

These new surfaces can be used for separation membranes [74], capillary electrophoresis, as blocking agents in ELISA tests or for different surface chemistries where non-specific adsorption of molecules needs to be inhibited and at the same time the immobilization of specific agents (antibodies, drugs, cells) has to be enhanced [75]. Surfactants can be basically divided into anionic (SDS: sodium dodecyl sulfate, PFOS: perfluorooctanesulfonate), cationic (DODAB: dioctadecyldimethylammonium bromide, CTAB: hexadecyl trimethyl ammonium bromide) and zwitterionic or amphoteric(betains, sulfobetains, CHAPS(3-[(3-cholamidopropyl)dimethylammonio]-1-propanesulfonate)) and non-ionic surfactants (Triton, Polysorbats). Non-ionic surfactants possess non-dissociable head groups, such as alcohol, phenol, ether, ester or amide. A majority of these surfactants are made hydrophilic by the presence of a polyethylene glycol chain and are referred to as polymeric surfactants or surface-active polymers. There exist two main types of polymeric surfactants in so-called “block” or “graft” configurations. The most used block polymer surfactants are copolymers of ethylene oxide and propylene oxide. Poly (ethylene oxide) (PEO) is known and one of the most used surfactants for the creation of non-fouling surfaces. There exist many different studies on attachment of PEO to various surfaces (PP, PE, PET, PTFE, Silica). The simple physical adsorption results in the formation of unstable layers with a high tendency to desorb from the surface. The effect has been especially described for layer molecular weight PEO surfactants [76]. 

## 4. Some Specific Illustrations Applied to Protein Storage

Coating of surfaces with non-charged hydrophilic polymers like polyethylene glycol (PEG) and poly(methyl methacrylate) (PMMA) have been found to reduce the protein adsorption due to the reduction of electrostatic forces and the hydrophobic interactions between the surface and the proteins in solutions [77,78,79,80]. 

**Figure 6:**
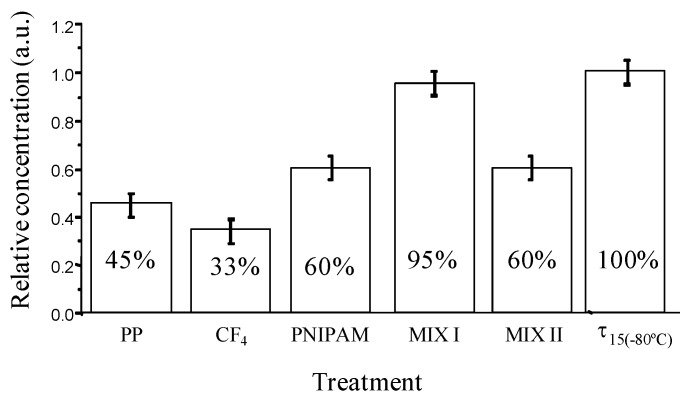
Relative concentration of PrPrec_hum_ stored for 24 h at 4 °C in supernatant solution (“sandwich” ELISA test) depending on surface chemistry of storage tubes.

New storage polypropylene tubes were designed with inner an surface either bearing hydrophilic or hydrophobic pendant groups and being either flat or nanostructured. These surfaces were elaborated from plasma treatment, grafting of polymers or mixtures of polymers (poly(N-isopropylacrylamide)) and surfactants (neutral PEO (20) sorbitan monolaurate (Tween 20), positively charged hexadecyltrimethylammonium bromide), called MIX I and MIX II, respectively. Their bioadhesion property (Figure 6) was checked with recombinant human prion protein (PrPrec_hum_) in PBS buffer solution, a biomarker of Creutzfeld Jacob disease [81,82].

Around 55% of the initial protein is lost due to the adsorption on untreated polypropylene tubes after 24 hours of storage. In the case of fluorinated tubes, the amount of protein adsorbed is even higher and reaches almost 70%. On the other hand, the storage of PrPrec_hum_ in hydrophilic Eppendorf tubes leads to an enhancement of the recovery (60%). The hydrophobic-hydrophobic interactions are strong enough to induce an irreversible proteinic adhesion while the protein deposition is somehow lower in the case of hydrophilic surfaces. Moreover, the other types of interactions such as electrostatic and surface texturation play an important role. Slightly better recovery of PrPrec_hum_ was observed from so-called MIX II tubes, nevertheless the loss of protein was still considerably high (40%). Opposed to this, so-called MIX I tubes disclosed low protein adhesion and high recovery. Only around 5% of the PrPrec_hum_ was adsorbed and 95% of protein remained in the solution. The efficiency of the non-adhesion property of the MIX I surfaces probably is associated to their relatively high hydrophilic, basic character and most importantly to an electrostatic repulsion between the protein and the surface, both positively charged in PBS. Structuration could also alter the deposition of proteins, since MIX I was shown to be more organized.

Another approach often used for implant devices is the immobilization of biological molecules like collagen or fibronectin. Therefore the modification of synthetic materials is directed in the development of surfaces having similar compositions to the extra cellular matrix (ECM), which comprises different proteins and polysaccharides [26,83].

## 5. Conclusions

For the future many materials can be used for different biomedical applications. Nevertheless, in order to satisfy the conditions of biocompatibility, their surface needs to be modified. The final prospect of the material will determine the properties of the surface and the strategy chosen for its modification. The modification routes have been shown to be strongly dependent on the biologic fluid nature and composition. Depending on the type of the biomolecule(s) and its (their) physicochemical properties, one specific surface modification must be chosen and in our knowledge, no universal surface elaboration has been applied up to this point.
